# Real-world efficacy of sequential nivolumab for metastatic renal cancer after first-line molecular targeting therapy

**DOI:** 10.1097/MD.0000000000029510

**Published:** 2022-08-12

**Authors:** Daisuke Obinata, Daigo Funakoshi, Fuminori Sakurai, Tsuyoshi Yoshizawa, Junichi Mochida, Kenya Yamaguchi, Satoru Takahashi

**Affiliations:** Department of Urology, Nihon University School of Medicine, Tokyo, Japan.

**Keywords:** mTOR inhibitor, nivolumab, renal cell carcinoma, tyrosine kinase inhibitor

## Abstract

This study aimed to clarify the real-world efficacy of sequential nivolumab for treating metastatic renal cancer after first-line molecular targeting therapy.

Patients were divided into two groups (2014–2016 and 2017–2020) according to the year when they started primary treatment with molecular targeted drugs (MTDs). We compared the overall survival of patients and investigated a contributing factor for survival.

The mean duration of overall survival was significantly longer in the 2017–2020 group (44.0 months) than in the 2014–2016 group (8.5 months). Univariate analysis showed that nivolumab treatment was a significant prognostic factor (*P* = .0021). Patients treated with nivolumab as second-line therapy had a significantly higher 5-year survival rate compared to that of other patients (70% vs 32%). In addition, the time from commencement of MTDs to switch to nivolumab was significantly shorter in the 2017–2020 group compared to the 2014–2016 group (8.94 vs 34.12 months, *P* = .03).

In our study, cases with first-line MTDs had markedly prolonged outcomes after the 2017 guideline update, and sequential nivolumab with prompt switching to nivolumab was an important factor.

## 1. Introduction

Renal cell carcinoma (RCC) is the seventh most frequently diagnosed tumor in the developed world and accounts for 1.8% of global cancer deaths.^[1]^ There are more than 10 histological subtypes of the disease, with clear cell RCC (ccRCC) being the most prevalent at 85%–90% of all RCC cases and accounting for the majority of cancer-related deaths.^[2]^ RCC is the deadliest of the urologic cancers, with a 5-year relative survival rate of 76% (2009–2015) in the U.S. Overall survival rates have improved significantly since the 1970s yet remain low at only 12% for stage IV metastatic disease.^[1]^ Initially, alterations of the Von Hippel Lindau (VHL) gene were found in a large number of ccRCC patients, and this was found to play an important role in the progression of RCC.^[3]^ It has been found that changes in VHL lead to the accumulation of hypoxia-inducible factor, resulting in increased expression of vascular endothelial growth factor receptor (VEGFR) and promotion of angiogenesis and proliferation. Therefore, tyrosine kinase inhibitors (TKIs) have been used that primarily target the VEGFR and platelet-derived growth factor receptor for the treatment of renal cancer. Although cytokine therapies showed 5 months of progression-free survival (PFS) and 21 months of overall survival, sunitinib in the first-line setting increased the median PFS to up to 11 months and overall survival to approximately 26 months.^[4]^ Following TKIs development, mammalian target of rapamycin (mTOR) pathway inhibitors have emerged and have shown similar results to TKIs.^[5]^ Moreover, with the emergence of immune checkpoint inhibitor (ICI) therapy that overtakes the outcomes of conventional first-line therapy using molecularly targeted drugs (MTDs), the treatment of metastatic RCC has entered a new era. Recently, the NCCN Kidney Cancer Panel discussed and updated the guidelines regarding initial management and first-line systemic therapy options using ICIs for patients with advanced clear cell RCC.^[6]^ It is expected that case studies of patients who received first-line MTDs will be underestimated in the future. However, there are still many cases in which MTDs are used as the first line, and although clinical trials have shown that the use of immune checkpoint inhibitors after MTD prolongs the prognosis,^[7]^ there are few reports that show the difference between real-world outcomes before and after the immune-oncology drug era. In this study, we retrospectively investigated the efficacy of updated guidelines in patients treated with first-line MTDs by comparing cases before and after nivolumab approval in Japan and examined whether important factors were affecting overall survival in these patients.

## 2. Materials and Methods

This study was approved by the Institutional Review Board and Research Ethics Committee of Nihon University School of Medicine (RK-190611-3 and RK-170711-7). Informed consent was obtained from all participants included in the study. In our institution, we treated 47 consecutive cases of metastatic renal cell cancer from January 2014 to October 2020. We retrospectively investigated 41 of the patients treated initially with TKIs or mTOR inhibitors and excluded six patients who received nivolumab and ipilimumab as first-line therapy. The dosing intervals and doses of each drug were within the standard of care and adjusted according to the patient’s condition at the discretion of the attending physician. None of the patients had previously received systemic anticancer therapy for advanced or metastatic disease. Patients were divided into two groups (2014–2016, 2017–2020) according to the year they started primary treatment with MTDs. Initial TNM classification, metastatic sites, the number of cases with primary tumor resection, IMDC risk classification, and the number of patients using each MTD and nivolumab in the respective groups were investigated as patient backgrounds. Changes in therapeutic agents were made at the discretion of the attending physician when side effects or clinical progression were observed and followed the guidelines in place at the time (e.g., TKI, mTOR; TKI, Nivolumab; mTOR, TKI). Overall survival from the start of first-line therapy was compared between the groups. In addition, second-line nivolumab (*n* = 11) and MTD patients (*n* = 11) were compared for Grade 2 or higher using the Common Terminology Criteria for Adverse Events ver 3.0, and overall response. Statistical analyses were performed using GraphPad Prism for Mac version 8 (GraphPad Software, Inc., La Jolla, CA) and JMP version 14 (SAS Institute Japan, Inc., Tokyo, Japan). Continuous data are presented as mean ± standard error. The Student’s *t* test was used to compare continuous data between groups. Chi-square and Fisher’s exact tests were used for categorical variables. Overall survival analyses were conducted according to the Kaplan–Meier method and survival characteristics were compared using the log-rank test. Univariate analysis was performed with each drug as a variable to determine its correlation with overall survival; a value <0.05 was considered statistically significant.

## 3. Results

**Figure 1.**
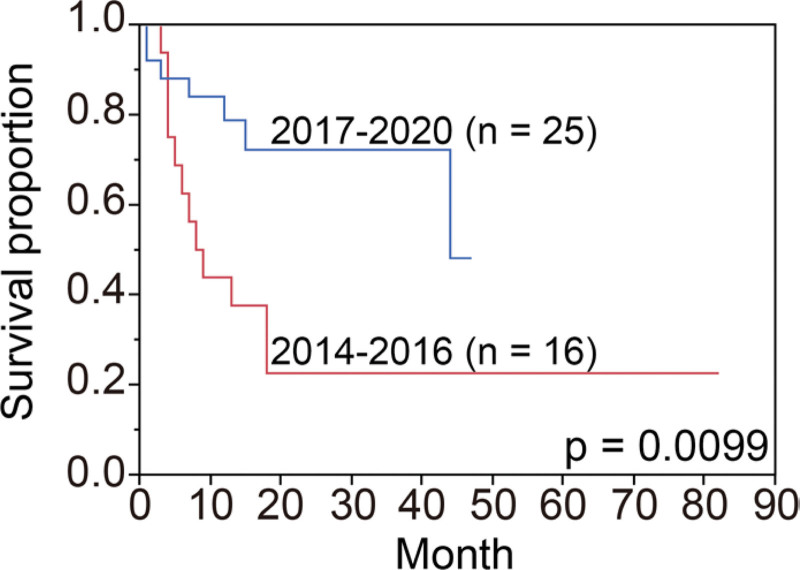
Kaplan-Meier estimates of overall survival with the year of treatment initiation stratified around 2017.

**Figure 2.**
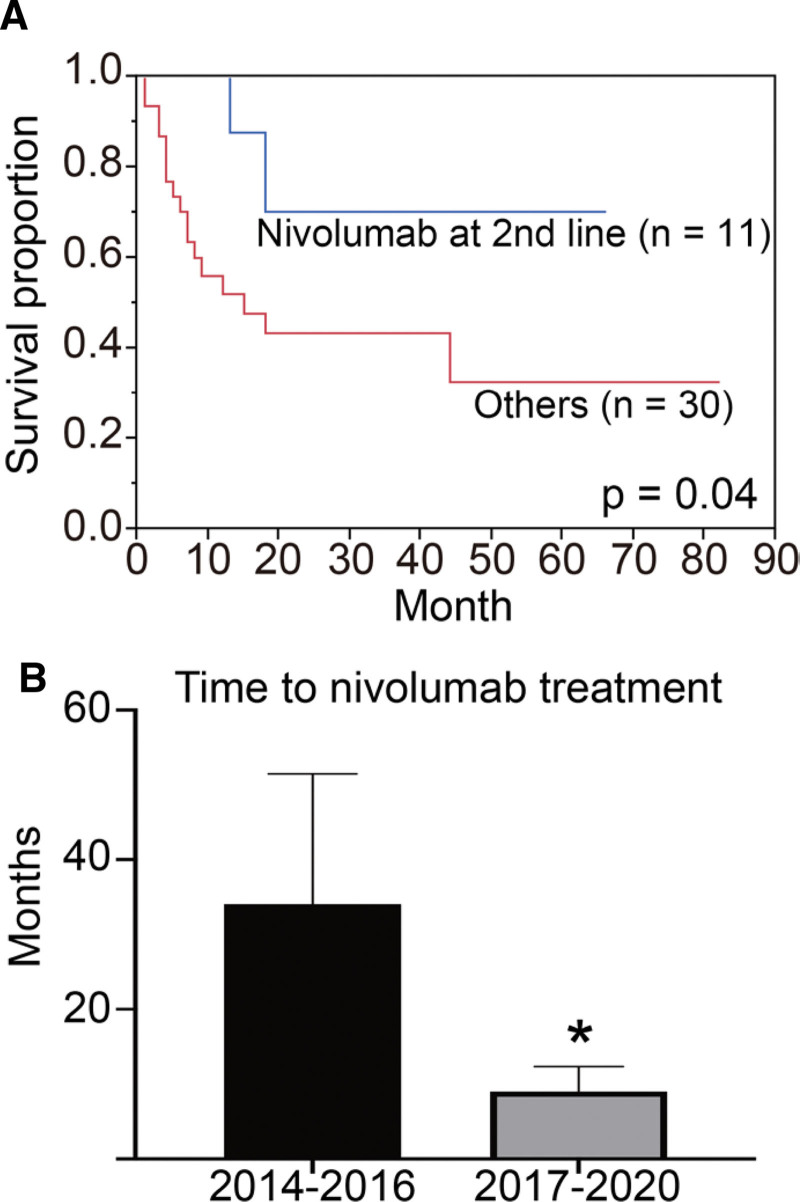
(A) Kaplan-Meier estimates of overall survival stratified using second-line treatment with nivolumab. (B) Comparison of time to nivolumab induction in each group (^*^, *P* < .05).

Of the 41 eligible patients, 16 underwent treatment between 2014 and 2016, and 25 between 2017 and 2020. In the 2014–2016 and 2017–2020 groups, the mean age was 65.8 and 67.8 years, respectively (Table 1, *P* = .52). There were no significant differences in the distribution of the groups in terms of TNM classification (T1: 31 vs 48%, T2: 6 vs 20%, T3: 56 vs 24%, T4: 6 vs 8%, *P* = .18; N1: 6 vs 12%, N2: 25 vs 16%, *P* = .68; M1: 56 vs 44%, *P* = .44), number of cases with primary tumor resection (56% vs. 64%, *P* = .62), and IMDC risk classification (favorable risk: 19% vs 16%, intermediate risk: 44% vs 60%, poor risk: 38% vs 24%, *P* = .56) (Table 1). The proportion of cases with liver metastases was slightly higher in the 2014–2016 group (*P* = .04, Table 1). The proportion of patients treated with axitinib was significantly lower in the 2017–2020 group (63% vs 28%, *P* = .028), whereas the number of patients treated with pazopanib was significantly higher in the 2017–2020 group (0 vs 24%, *P* = .01) (Table 1). Notably, the proportion of nivolumab did not differ between groups. The mean duration of overall survival was longer in the 2017–2020 group (44.0 months) than in the 2014–2016 group (8.5 months), with a death hazard ratio of 0.35 (95% CI, 0.13–0.92) (Fig. 1). Univariate analysis showed that nivolumab treatment was significantly associated with a good prognosis, whereas MTD treatment was not (HR 5.26, 95% CI 1.74–22.71, *P* = .0021; Table 2). Investigating the effect of sequential administration of nivolumab, a significantly higher 5-year survival rate was observed in patients who received nivolumab as second-line therapy than in other patients (70% vs 32%, *P* = .04) (Fig. 2A). Patients with second line use of nivolumab showed a significantly lower AE compared to second line use of MTDs, excluding those who had first line treatment terminated (*n* = 19) and no PD in maximum response was observed (Table 3). In addition, the time from commencement of MTDs to switch to nivolumab was significantly shorter in the 2017–2020 group compared to the 2014–2016 group (8.94 vs 34.12 months, *P* = .03) (Fig. 2B). Of the 16 patients treated with nivolumab, 7 (3 in the 2014–2016 group, 4 in the 2017–2020 group, *P* = .14) received an MTD after the termination of nivolumab. Axitinib was the most frequent treatment after nivolumab was completed, followed by cabozantinib (Table 4). In the cases with MTD-nivolumab-MTD sequence, we observed no significant difference in the mean duration of MTD administration before and after nivolumab treatment (Table 4, *P* = .24).

**Table 1:** Patient characteristics of each group. Student’s *t* test was used to compare the average age between groups.

	2014–2016 (*n*0 =0 16)	2017–2020 (*n*0 =0 25)	*P*
The average age at initiation of treatment for metastasis (S.D.)	65.8 (9.20)	67.8 (10.36)	.52
Initial TNM classification			
T1	5	12	.18
T2	1	5	
T3	9	6	
T4	1	2	
N1	1	3	.68
N2	4	4	
M1	9	11	.44
Metastatic sites			
Lymph node	5	13	.19
Bone	8	7	.15
Lung	9	13	.78
Liver	4	1	.04
The others	4	4	.48
Number of cases with resected primary tumors	9	16	.62
IMDC risk classification			
Favorable risk	3	4	.56
Intermediate risk	7	15	
Poor risk	6	6	
Number of cases treated by each drug			
Everolimus	1	3	.53
Sunitinib	9	14	.98
Axitinib	10	7	.028
Pazopanib	0	6	.01
Sorafenib	2	3	.96
Nivolumab	4	12	.13
Cabozantinib	1	2	.83

**Table 2:** Univariate proportional hazard analyses were performed with each drug as a variable.

Variable	Hazard ratio	95% CI	*P*
Everolimus	3.17	0.64–57.3	.18
Sunitinib	0.97	0.38–2.43	.95
Axitinib	1.00	0.40–2.55	.99
Pazopanib	1.46	0.41–9.25	.59
Sorafenib	0.34	0.13–1.09	.06
Nivolumab	5.26	1.74–22.71	.0021
Cabozantinib	1.1	0.30–7.14	.89

**Table 3:** The result of 2nd line treatment.

	Nivolumab (*n*0 =0 11)	MTDs (*n*0 =0 11)	*P*
Maximal response			.09
CR	1	1	
PR	1	2	
SD	9	4	
PD	0	4	
Adverse event (>CTCAE grade 2)			
Number	3	8	.03
Anal Fistula	0	1	
Cerebral infarction	0	1	
Diarrhea	1	1	
Thyroid dysfunction	1	1	
Hypertension	0	1	
Ileus	0	1	
Renal dysfunction	0	1	
Stomatitis	0	1	

**Table 4:** Characteristics of MTDs before and after treatment with nivolumab (*n* = 7).

	Before	After
Number of cases treated by each drug		
Everolimus	2	1
Sunitinib	2	0
Axitinib	5	5
Pazopanib	1	0
Sorafenib	0	0
Cabozantinib	0	3
The average duration of treatment (months)	8.9	6.4

Chi-squared test was used for analyzing the N and M parameters in the TNM classification, each parameter in metastatic sites, number of cases with resected primary tumors and number of cases treated by each drug. Fisher’s exact tests were used for other categorical variables.</p>IMDC = international metastatic RCC database consortium, SD = standard deviation

The cases wherein first-line treatment with MTD was terminated were excluded (*n* = 19). Fisher’s exact tests were performed for each variable.CR = complete response, CTCAE = common terminology criteria for adverse events, MTDs = molecular targeted drugs, PD = progressive disease, PR = partial response, SD = stable disease.

## 4. Discussion

In this study, we examined the actual prolongation of overall survival by the updated treatment guidelines for patients receiving first-line MTD treatment by comparing the results of cases before and after the introduction of ICI treatment. Our data showed a significant increase in overall survival in patients who started first-line MTDs after 2017, with nivolumab being an important contributing factor.

The Japanese guidelines for the treatment of advanced renal cancer have also changed over the years.^[8]^ First, the Japanese guidelines were updated in 2017, characterized by a change from the MSKCC classification to the IMDC risk classification for clear cell carcinoma and the recommendation of nivolumab as a second-line treatment following MTDs. In 2019, combination therapy with ICIs was recommended as the first-line treatment for intermediate and poor-risk patients, and in 2020, combination therapy with ICIs plus TKI and cabozantinib alone was recommended as the first-line treatment. Based on these changes, it is expected that there will be a shift to ICI-based therapy in the future. However, TKIs are still recommended in the current guidelines as first-line therapy; therefore, to evaluate recommendations, it is important to accumulate long-term follow-up data.

Interestingly, there was a significant decrease in the number of patients receiving axitinib treated after 2017. Since axitinib was the most common option after nivolumab treatment, this might be indicative of an increase in the number of patients choosing nivolumab followed by axitinib. In our data, there was no significant difference in the duration of TKI response before and after nivolumab treatment, suggesting that nivolumab has less unfavorable post-treatment effects. Axitinib was approved as a second-line treatment for advanced RCC by the US and European institutions in 2012 after failing to show efficacy in first-line treatment compared to sorafenib.^[9]^ Although comparative studies of everolimus and nivolumab in second-line therapy have been reported,^[10]^ Japanese renal cancer guidelines issued in 2017 mention that there are no large studies directly comparing axitinib and nivolumab as second-line treatment.^[8]^ There was no difference in our case in the distribution of IMDC risk classification between second-line nivolumab and axitinib patients (data not shown). Furthermore, two of the seven cases were treated with cabozantinib, and these patients tended to have a relatively longer duration of treatment with other MTDs after nivolumab treatment. Cabozantinib targets the resistance induced by other TKIs and nivolumab.^[11–13]^ Our data showed that second-line nivolumab significantly prolonged overall survival, suggesting that nivolumab might be more effective than MTDs as second-line therapy. Despite no significant difference in the number of cases treated with nivolumab between the 2014–2016 and 2017–2020 groups, overall survival was significantly longer in the 2017–2020 group. In contrast, the time to introduce nivolumab was significantly shorter in the 2017–2020 group. These data indicate that not only the use of nivolumab in second-line treatment but also prompt switching to nivolumab has an impact on overall survival.

This study has several limitations including its retrospective nature, a low number of patients, and single racial factors (only Japanese cases were evaluated). In addition, since the evidence was obtained at a single university hospital, there could be referral bias. Follow-up bias may be unavoidable with patients being transferred home for subsequent care at the end of lives, depending on their circumstances. Despite this, our study has illustrated the impact of guideline revision on the treatment efficacy of the first TKI for metastatic renal cancer in Japan.

## 5. Conclusion

In our study, cases with first-line MTDs had markedly prolonged outcomes after the 2017 guideline update, and sequential nivolumab was an important factor.

## Acknowledgments

The authors thank Ms. Naoko Kodaka for her secretarial assistance. We would like to thank Editage (www.editage.com) for English language editing.

## Author contributions

Conceptualization: Daisuke Obinata, Kenya Yamaguchi, Satoru Takahashi.

Data curation: Daigo Funakoshi, Fuminori Sakurai, Junichi Mochida, Tsuyoshi Yoshizawa.

Funding acquisition: Daisuke Obinata.

Investigation: Kenya Yamaguchi, Satoru Takahashi.

Methodology: Daisuke Obinata, Satoru Takahashi.

Project administration: Daisuke Obinata, Kenya Yamaguchi, Satoru Takahashi.

Supervision: Kenya Yamaguchi, Satoru Takahashi.

Writing – original draft: Daisuke Obinata.

Writing – review & editing: Kenya Yamaguchi.
